# Supplementary material for: Time-dependent assessment of stimulus-evoked regional dopamine release
Source: Nat Commun. 2019 Jan 18;10:336. doi: 10.1038/s41467-018-08143-4 (PMC6338792; doi:10.1038/s41467-018-08143-4)
Supplement: Supplementary file 1 — Supplementary Information [file 41467_2018_8143_MOESM1_ESM.pdf]

Supplementary Information: Time-dependent assessment of stimulus-evoked regional dopamine release

Rachel N. Lippert[1,#], Anna Lena Cremer [1,#], Sharmili Edwin Thanarajah[1,2], Clio Korn[3,4], Thomas Jahans-Price[4], Lauren M. Burgeno[4], Marc Tittgemeyer[1,5,6], Jens C. Brüning[1,5,7], Mark E. Walton[4,8], Heiko Backes[1,*]

[1]Max Planck Institute for Metabolism Research, Gleueler Str. 50, 50931 Cologne, Germany

[2]Department of Neurology, University Hospital of Cologne, Kerpener Str. 62, 50937 Cologne, Germany

[3]Department of Psychiatry, University of Oxford, Warneford Hospital, Oxford OX3 7JX, U.K.

[4]Department of Experimental Psychology, University of Oxford, Tinsley Building, Mansfield Road, Oxford OX1 3SR, U.K.

[5]Cologne Cluster of Excellence in Cellular Stress and Aging-Associated Disease (CECAD), Joseph-Stelzmann-Str. 26, 50931 Cologne, Germany

[6]Modern Diet and Physiology Research Center, 290 Congress Avenue, New Haven, CT 06519, USA

[7]Center for Endocrinology, Diabetes and Preventive Medicine (CEPD), University Hospital of Cologne, Kerpener Str. 62, 50937 Cologne, Germany

[8]Wellcome Centre for Integrative Neuroimaging, Department of Experimental Psychology, University of Oxford, Tinsley Building, Mansfield Road, Oxford OX1 3SR, UK.

Content

1. Supplementary Notes
2. Supplementary Discussion
3. Supplementary Figures
4. Supplementary References

1. Supplementary Notes

Behavioral testing of $hM3D_{Gq}^{DAT}$ mice

At baseline, control and transgenic animals displayed no differences in body weight ($hM3D_{Gq}^{WT}$: 22.91g±1.23 vs. $hM3D_{Gq}^{DAT}$: 22.21g±1.61, p=0.19) nor did they display any differences in normal behavior.

After CNO injection, $hM3D_{Gq}^{DAT}$ mice displayed increased locomotor behavior, including an overall increase in distance traveled, stereotypic behaviors and ambulatory episodes indicative of dopaminergic activation, all of which return to baseline levels after two hours (genotype x treatment interaction: $F(1,28)_{distance\ traveled}$=5.248, p=0.0297; $F(1,28)_{stereotypic\ behaviors}$=4.916, p=0.0349; $F(1,28)_{ambulatory\ episodes}$=3.557, p=0.0697, n=7-10, Three Way ANOVA) (Figure 3A-F). CNO injection additionally increased rotational behavior specifically in $hM3D_{Gq}^{DAT}$ mice (total turns±SEM: $hM3D_{Gq}^{WT}$+Saline=54.57±2.82, $hM3D_{Gq}^{WT}$+CNO=82.71±13.27, $hM3D_{Gq}^{DAT}$+Saline=95.75±14.59, $hM3D_{Gq}^{DAT}$+CNO=201.0±15.74; genotype x treatment interaction: $F(1,28)_{total\ turns}$=7.772, p=0.0094; $F(1,28)_{clockwise\ turns}$=4.749, p=0.0379, $F(1,28)_{counterclockwise\ turns}$ =3.529, p=0.0708, n=7-10, Three Way ANOVA) (Figure 3G, see additional discussion in the Supplemental Results). Neither CNO nor saline injection into $hM3D_{Gq}^{WT}$ mice induced significant behavioral effects (Figure 3A-G). Therefore we used $hM3D_{Gq}^{DAT}$ mice in the subsequent PET studies, to allow for pairwise comparison of baseline and stimulated responses within genotype.

Hemispheric differences of DA release in $hM3D_{Gq}^{DAT}$ mice

After injection of CNO in $hM3D_{Gq}^{DAT}$ mice we found a significant increase of DA release in the left striatum in terms of increased temporal variations of the [11C]raclopride signal (Figure 7). The increase in the right striatum was less pronounced (Supplementary Figure 3). This observation is consistent with behavioral data of these mice that show increased clockwise rotation after activation with CNO (Figure 3G). The preference for clockwise rotation was reported to be linked to higher DA levels in the left striatum [1,2]. Furthermore, in our PET studies, 4/5 animals showed a higher increase in rDA in the left striatum. This closely corresponds to human literature, with right-handed dominance being linked to high DA in the left striatum. Therefore in the human studies, only right-handed individuals are selected for monitoring, which would limit the amount of variability between hemispheres between subjects, selecting for individuals which likely have a left striatum dominance.

2. Supplementary Discussion

In this study we provide evidence that rapid changes of DA activity can be indirectly measured *in vivo* by using temporal variations of the [11C]raclopride PET signal as a surrogate signal. Noise, inherently present in measured data, also contributes to the temporal variations assessed by *rDA*. However, application of the same measurement protocol (same amount of tracer, timing, PET gantry, etc.) within a study ensures that the contribution of noise is the same in each measurement. The contribution of noise to *rDA* is related to the signal strength and increases steadily in time due to radioactive decay of [11C]raclopride (half life = 20 minutes). This effect is presumably responsible for the positive trend in all *rDA* data, mouse and human.

Using a transgenic mouse model, we engineered mice that cells expressing the dopamine transporter also express the excitatory DREADD, $hM3D_{Gq}$, via Cre/loxP-mediated recombination. DREADDs were activated by intraperitoneal injection of the drug CNO [3]. Recently it was reported that CNO does neither cross the blood brain barrier nor bind to DREADD receptors with high affinity but that its metabolite, clozapine, instead is a potent agonist to $hM3D_{Gq}$ and binds with much higher affinity to the DREADD than it binds to its endogenous receptors [4]. Since we observe clear activations after injection of 0.3 mg/kg (body weight) of CNO, we revisited our results to determine if they comply with this report. Raper et al. showed that 10 minutes after subcutaneous injection of CNO into non-human primates the clozapine level in the plasma rises to ~1% of the CNO plasma level which in our case would correspond to 0.003 mg/kg clozapine [5]. Gomez et al. report effects at a dose of 0.01 mg/kg clozapine in mice that carry a $hM4D_{Gi}$ receptor, which – according to their results – has a ~4 times lower affinity for clozapine than $hM3D_{Gq}$ that we used in our model. Taking this into account, we would expect activation of the DREADDs at the dose of CNO we injected into the mice. Gomez et al. also report that no effects from clozapine binding to endogenous receptors were observed at this dose. However, in our study the primary aim was to simply activate the dopaminergic system reliably. For this purpose the precise mechanism of activation was less important.

Our method for the detection of DA release with [11C]raclopride PET requires steady state conditions. It can therefore be applied to all data that were acquired with a bolus plus constant infusion injection of the tracer. Data should then be rebinned into timeframes of equal duration. The duration of the timeframes must be chosen to be sufficiently long enough to provide a good signal-to-noise ratio even at the end of the scan time. Depending on the type of PET gantry used, we recommend a timeframe duration of five minutes. *rDA* can be directly calculated from the PET data by application of Equation 10 and 11, which can be easily

performed with any type of script language or even manually. No step of data fitting to a model is necessary. Furthermore, it is important that the conditions are similar in each PET session (injected tracer activity, temperature, etc.) in order to increase comparability of regional *rDA* between different subjects.

There are controversies about extrastriatal binding of raclopride. The density of D2Rs in the extrastriatal regions we analyzed is known to be 2%-8% of that in the striatum [6]. Although being lower than in striatum we would still expect that a percentage of the [11C]raclopride is specifically bound to these receptors. Accordingly, after 60 minutes the uptake of [11C]raclopride (specific plus non-specific) in the cortex is 3-4 times lower than in the striatum [7]. Several groups observed task-related or pharmacologically-induced changes of the absolute raclopride signal in extrastriatal regions [8–10]. However, other groups using high-affinity radioligands that are more suitable for imaging of extrastriatal D2 receptors did not detect changes in tracer binding after amphetamine injection [11,12]. Using the method described here, temporal variations in the signal relative to the total signal were analyzed instead of the absolute [11C]raclopride uptake, which makes the method more sensitive for the detection of variations in regions with low absolute binding in comparison to methods that analyze stimulation-related changes in the absolute signal. Another property of the DA system promotes the detection of DA release in terms of minute-by-minute variations in extrastriatal regions: although less DA is released in extrastriatal regions due the lower density of DA synapses, the major fraction of released DA is removed at a minute time scale due to the lower density of DATs. This means that the amplitudes of release-induced minute-by-minute variations in extracellular DA concentrations are of the same order of magnitude in extrastriatal regions as in the striatum despite the difference in total amount of released DA (Figure 2). [13] From these considerations and the fact that the regions that are highlighted in the human PET study are well-known to be involved in reward processing, temporal variations of the [11C]raclopride signal appear to be a valuable measure for DA release even in extrastriatal regions. Tracers with higher affinities for DA receptors provide higher extrastriatal signals but are less sensitive to variations of endogenous DA levels.

3. Supplementary Figures

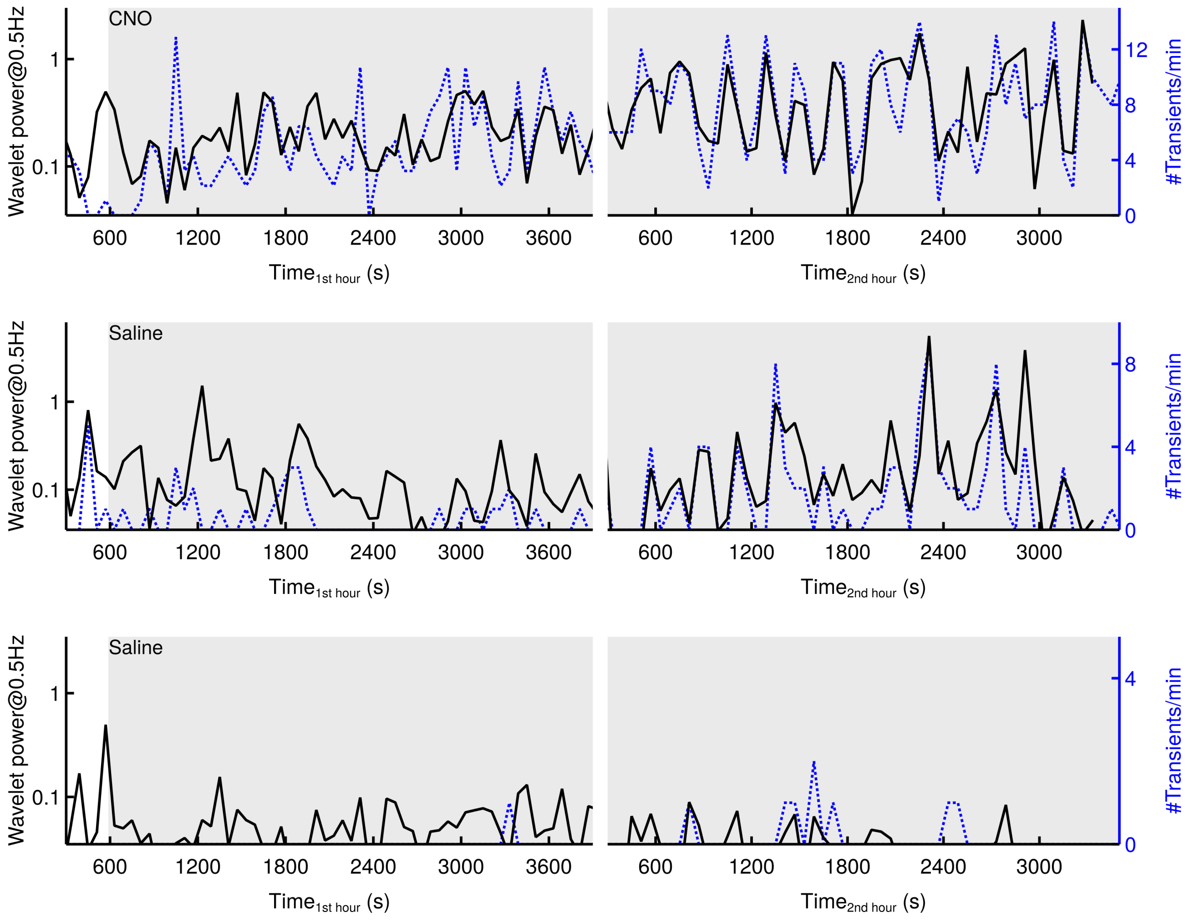

Supplementary Figure 1. Transient rates and wavelet power at 0.5 Hz in individual $hM3D_{Gq}^{DAT}$ mice after CNO/saline injection

Wavelet power at 0.5 Hz (black line) and transient rates (dashed blue line) in one-minute intervals during the first (left) and second (right) block of continuous FSCV recording in three individual mice. The grey box indicates the time after CNO/Saline injection. While there is remarkable synchronism between transient rates and 0.5 Hz wavelet power in the first two mice, the agreement is less in the last mouse, presumably due to a failure of the automatized detection of transient rates.

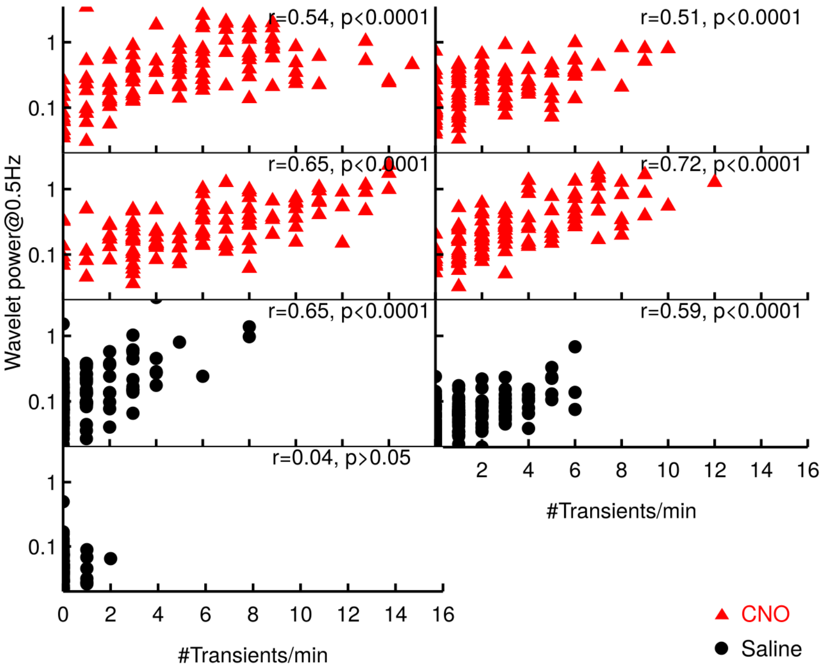

Supplementary Figure 2. Correlations between transient rates and wavelet power at 0.5 Hz in individual animals

Wavelet power at 0.5 Hz and transients rates of the individual $hM3D_{Gq}^{DAT}$ mice. Wavelet power at 0.5 Hz and transient rates in one-minute intervals correlated significantly with Pearson correlation coefficient $r>0.5$ with the exception of the last mouse (red triangles=$hM3D_{Gq}^{DAT}$+ CNO; black circles=$hM3D_{Gq}^{DAT}$+ Saline).

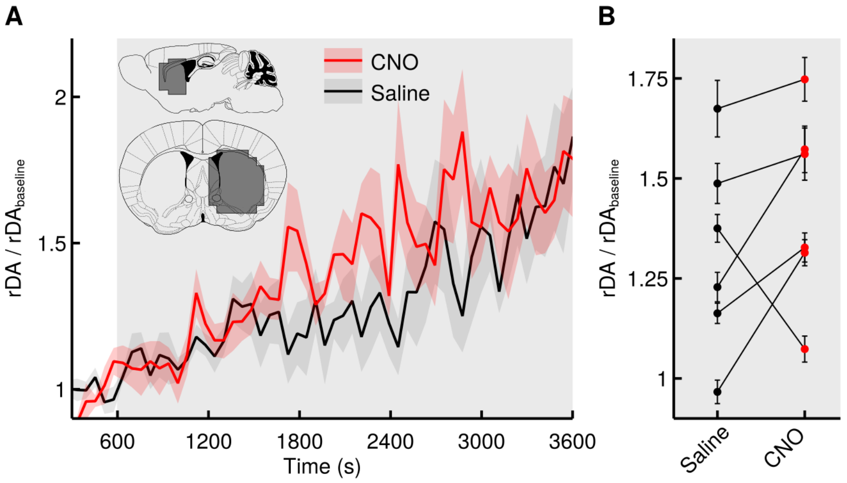

Supplementary Figure 3. Dopamine release assessed by [11C]raclopride PET in hM3D$_{Gq}^{DAT}$ mice in the right striatum

(A) Dopaminergic activity changes temporal variations of the [11C]raclopride signal (rDA, mean±SEM). In n=6 mice rDA was increased in the right striatum after chemogenetic activation with CNO (red line) in comparison to vehicle injection with saline (black line) at t=600 s. The grey background indicates the time after injection. (B) Average *rDA* after t=600 s in each mouse after saline and CNO injection. Increase of *rDA* after CNO injection was not significant (Paired Student's *t*-test, n=6, t=1.197, df=5, p=0.285).

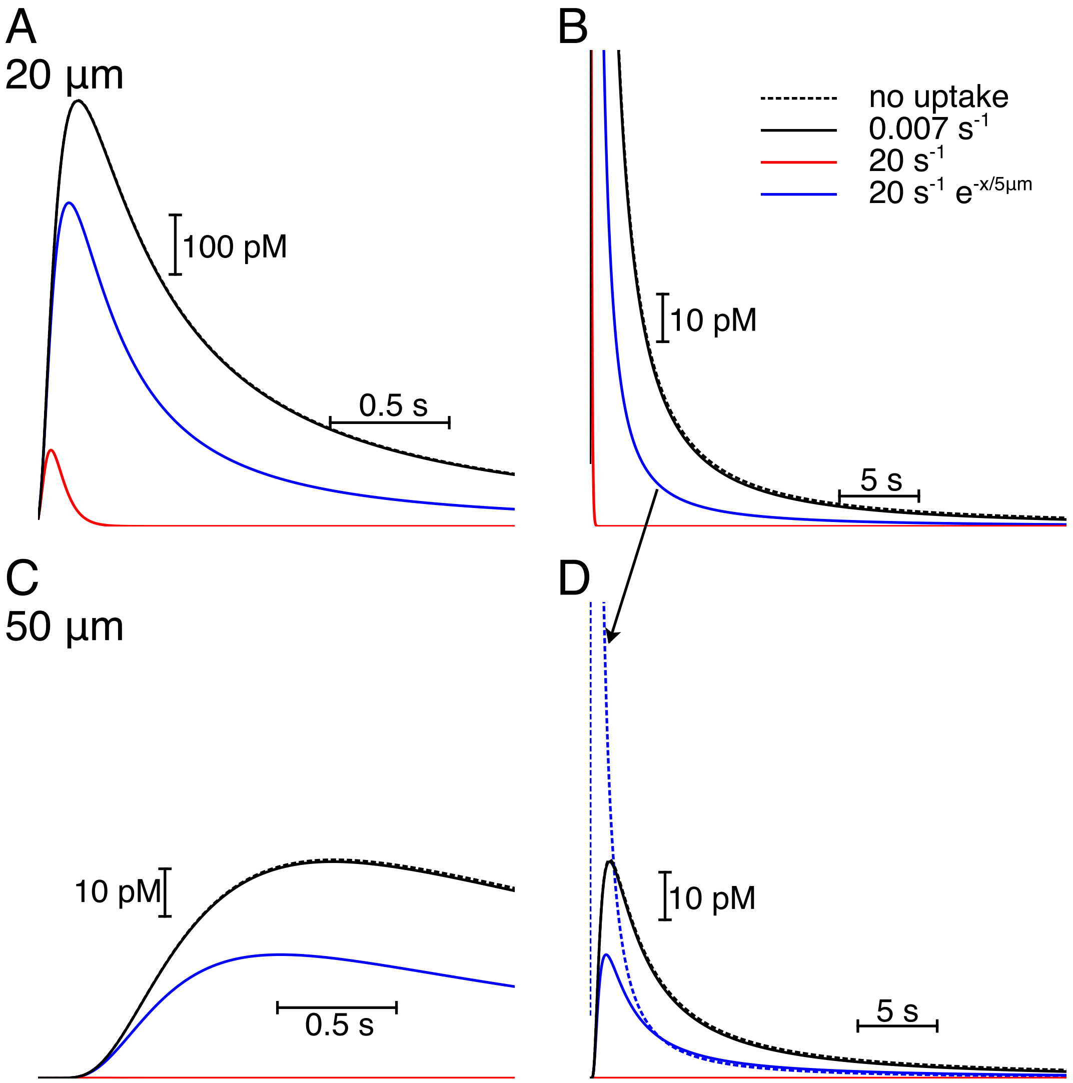

Supplementary Figure 4. Dopamine transients after quantal release in tissue with heterogeneous DAT expression

The model for quantal release of 9800 DA molecules is identical to that used by Cragg and Rice [14] with the difference that the differential equation is solved numerically instead of using the analytical solution. In this way the model can be calculated for any spatial distribution of DATs. (A) Extracellular DA concentration during the first two seconds after quantal release at a distance of 20 µm from the release site. The traces for high (black) and low (red) homogenous DAT expression are identical to those in Figure 1 of Cragg and Rice. Assuming exponential decrease of DAT expression as a function of distance from the release site reduces the peak height (blue). (B) Extracellular DA concentration during the first 30 seconds after quantal release. If DA kinetics is determined by diffusion only (no uptake), a noticeable fraction of DA is cleared at a minute time scale while at high homogenous DAT expression all DA is removed within 0.5 seconds (red). A small but noticeable minute time scale component is also present at exponential decrease of DAT expression from the release site (blue). C and D show extracellular DA concentrations 50 µm from the release site. While the

fast component, that determines the peak, critically depends on the distance from the release site, the minute time scale slow component is nearly identical at 20 μm and 50 μm distance.

4. Supplementary References

1. Molochnikov, I. & Cohen, D. Hemispheric differences in the mesostriatal dopaminergic system. *Front. Syst. Neurosci.* **8,** 110 (2014).
2. Glick, S. D., Jerussi, T. P., Water, D. H. & Green, J. P. Amphetamine-induced changes in striatal dopamine and acetylcholine levels and relationship to rotation (circling behavior) in rats. *Biochem. Pharmacol.* **23,** 3223–3225 (1974).
3. Armbruster, B. N., Li, X., Pausch, M. H., Herlitze, S. & Roth, B. L. Evolving the lock to fit the key to create a family of G protein-coupled receptors potently activated by an inert ligand. *Proc. Natl. Acad. Sci. U. S. A.* **104,** 5163–5168 (2007).
4. Gomez, J. L. *et al.* Chemogenetics revealed: DREADD occupancy and activation via converted clozapine. *Sci. (New York, NY)* **357,** 503–507 (2017).
5. Raper, J. *et al.* Metabolism and Distribution of Clozapine-N-oxide: Implications for Nonhuman Primate Chemogenetics. *ACS Chem. Neurosci.* **8,** 1570–1576 (2017).
6. Suhara, T. *et al.* Extrastriatal dopamine D2 receptor density and affinity in the human brain measured by 3D PET. *Int. J. Neuropsychopharmacol.* **2,** 73–82 (1999).
7. Alakurtti, K. *et al.* Long-Term Test–Retest Reliability of Striatal and Extrastriatal Dopamine D 2/3Receptor Binding: Study with [11C]Raclopride and High-Resolution PET. *J. Cereb. Blood Flow Metab.* **35,** 1199–1205 (2015).
8. Piccini, P., Pavese, N. & Brooks, D. J. Endogenous dopamine release after pharmacological challenges in Parkinson's disease. *Ann. Neurol.* **53,** 647–653 (2003).
9. Sawamoto, N. *et al.* Cognitive deficits and striato-frontal dopamine release in Parkinson’s disease. *Brain* **131,** 1294–1302 (2008).
10. Stokes, P. R. A. *et al.* Significant decreases in frontal and temporal [11C]-raclopride binding after THC challenge. *Neuroimage* **52,** 1521–1527 (2010).
11. Slifstein, M. *et al.* Striatal and extrastriatal dopamine release measured with PET and [(18)F] fallypride. *Synap. (New York, NY)* **64,** 350–362 (2010).
12. Aalto, S. *et al.* The effects of d-amphetamine on extrastriatal dopamine D2/D3 receptors: a randomized, double-blind, placebo-controlled PET study with [11C]FLB 457 in healthy subjects. *Eur.*

J. Nucl. Med. Mol. Imaging **36,** 475–483 (2009).

13. Garris, P. A., Collins, L. B., Jones, S. R. & Wightman, R. M. Evoked Extracellular Dopamine In Vivo in the Medial Prefrontal Cortex. *J. Neurochem.* **61,** 637–647 (2006).

14. Cragg, S. J. & Rice, M. E. DAncing past the DAT at a DA synapse. *Trends Neurosci.* **27,** 270–277 (2004).
