# Supplementary material for: Time-dependent assessment of stimulus-evoked regional dopamine release
Source: Nat Commun. 2019 Jan 18;10:336. doi: 10.1038/s41467-018-08143-4 (PMC6338792; doi:10.1038/s41467-018-08143-4)
Supplement: Supplementary file 3 — Reporting Summary [file 41467_2018_8143_MOESM3_ESM.pdf]

nature research

Corresponding author(s): Heiko Backes

Last updated by author(s): Dec 14, 2018

Reporting Summary

Nature Research wishes to improve the reproducibility of the work that we publish. This form provides structure for consistency and transparency in reporting. For further information on Nature Research policies, see Authors & Referees and the Editorial Policy Checklist.

Statistics

For all statistical analyses, confirm that the following items are present in the figure legend, table legend, main text, or Methods section.

n/a	Confirmed	
☐	☒	The exact sample size (*n*) for each experimental group/condition, given as a discrete number and unit of measurement
☐	☒	A statement on whether measurements were taken from distinct samples or whether the same sample was measured repeatedly
☐	☒	The statistical test(s) used AND whether they are one- or two-sided<br>*Only common tests should be described solely by name; describe more complex techniques in the Methods section.*
☒	☐	A description of all covariates tested
☐	☒	A description of any assumptions or corrections, such as tests of normality and adjustment for multiple comparisons
☐	☒	A full description of the statistical parameters including central tendency (e.g. means) or other basic estimates (e.g. regression coefficient) AND variation (e.g. standard deviation) or associated estimates of uncertainty (e.g. confidence intervals)
☐	☒	For null hypothesis testing, the test statistic (e.g. *F*, *t*, *r*) with confidence intervals, effect sizes, degrees of freedom and *P* value noted<br>*Give P values as exact values whenever suitable.*
☒	☐	For Bayesian analysis, information on the choice of priors and Markov chain Monte Carlo settings
☒	☐	For hierarchical and complex designs, identification of the appropriate level for tests and full reporting of outcomes
☒	☐	Estimates of effect sizes (e.g. Cohen's *d*, Pearson's *r*), indicating how they were calculated

Our web collection on statistics for biologists contains articles on many of the points above.

Software and code

Policy information about availability of computer code

Data collection	Siemens IAW 2.0.0.1050, Siemens HRRT Scan Control Software V 1.1.0.0608, Tarheel CV (National Instruments), Med Associates, Activity Monitor 7 Software, JASP Team (2018), JASP (Version 0.9)
Data analysis	GraphPad Prism 7.0d, Matlab 2014, VINCI 4.9, IDL 8.5.1, Gcc 4.8.4, Numerical Recipes in C, 3rd edition, Veusz 1.18, R 2.15.3

For manuscripts utilizing custom algorithms or software that are central to the research but not yet described in published literature, software must be made available to editors/reviewers. We strongly encourage code deposition in a community repository (e.g. GitHub). See the Nature Research guidelines for submitting code & software for further information.

Data

Policy information about availability of data

All manuscripts must include a data availability statement. This statement should provide the following information, where applicable:

- Accession codes, unique identifiers, or web links for publicly available datasets
- A list of figures that have associated raw data
- A description of any restrictions on data availability

The data that support the findings of this study are available from the corresponding author upon reasonable request.

Field-specific reporting

Please select the one below that is the best fit for your research. If you are not sure, read the appropriate sections before making your selection.

☒ Life sciences ☐ Behavioural & social sciences ☐ Ecological, evolutionary & environmental sciences

October 2018

Life sciences study design

All studies must disclose on these points even when the disclosure is negative.

Sample size	The sample size for PET scans was determined based on previous literature using amphetamine to stimulate dopamine release (Rice et al Neuropsychopharmacology 2001). For FSCV studies, initial sample sizes were based on Syed et al (Nature Neuroscience 2016). For behavioral analyses, group sizes were based on findings by Wang et al (Neuroscience Bulletin 2013) using viral injection of hM3DGq and CNO to stimulate locomotor responses.
Data exclusions	No animals were excluded from behavioral analysis. In PET studies, only animals receiving both treatments successfully were used in the final analyses. In the voltammetry data, one vehicle injected animal was excluded from baseline statistical analysis due to lack of any detectable transients (as would be expected without stimulation).
Replication	Replicate experiments were successful.
Randomization	In behavioral experiments, littermate mice were randomly assigned to treatment group and were matched for age. For PET studies, mice received both CNO and saline injections in a balanced approach, with some receiving CNO first followed by Saline and vice versa. In FSCV experiments, animals were randomly assigned to treatment group and age-matched where possible.
Blinding	Experimenters were not blinded to genotypes during behavioral experiments. In PET and FSCV experiments, only one genotype was used for experiment type so not blinding was possible.

Reporting for specific materials, systems and methods

We require information from authors about some types of materials, experimental systems and methods used in many studies. Here, indicate whether each material, system or method listed is relevant to your study. If you are not sure if a list item applies to your research, read the appropriate section before selecting a response.

Materials & experimental systems

n/a	Involved in the study	
☒	☐	Antibodies
☒	☐	Eukaryotic cell lines
☒	☐	Palaeontology
☐	☒	Animals and other organisms
☐	☒	Human research participants
☒	☐	Clinical data

Methods

n/a	Involved in the study	
☒	☐	ChIP-seq
☒	☐	Flow cytometry
☒	☐	MRI-based neuroimaging

Animals and other organisms

Policy information about studies involving animals; ARRIVE guidelines recommended for reporting animal research

Laboratory animals	Mice: C57BL/6N background and hM3DGqDAT mice (see methods), male, 13-37 weeks
Wild animals	The study did not involve wild animals.
Field-collected samples	N/A
Ethics oversight	Bezirksregierung Köln, University of Oxford ethical review board

Note that full information on the approval of the study protocol must also be provided in the manuscript.

Human research participants

Policy information about studies involving human research participants

Population characteristics	10 healthy, male, normal-weight (BMI: 25.73 ± 2.67, age: 57.1 ± 10.55) and non-smoking volunteers recruited from a preexisting database of Max Planck Institute for Metabolism Research. No history of neurological, psychiatric or eating disorders was present. Further exclusion criteria were special diets, lactose intolerance, diabetes, the participation in a previous PET study and a score higher than 12 in the Beck Depression Inventory (BDI II).
Recruitment	Database of Max Planck Institute for Metabolism Research
Ethics oversight	Local ethics committee of the Medical Faculty of the University of Cologne (Cologne, Germany)

Note that full information on the approval of the study protocol must also be provided in the manuscript.
